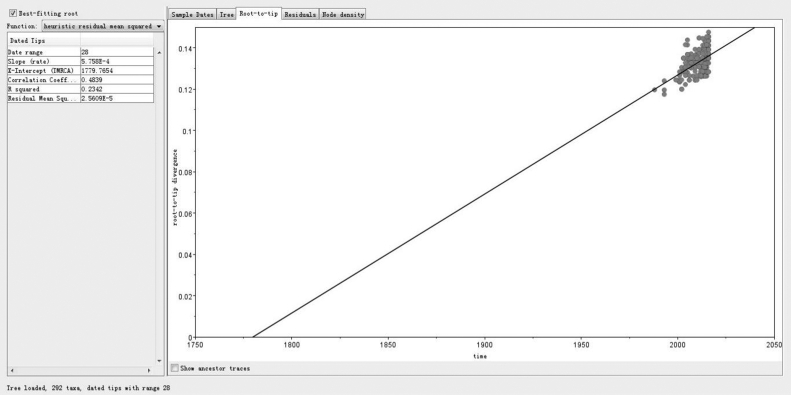# Genotype distribution and evolutionary analysis of rotavirus associated with acute diarrhea outpatients in Hubei, China, 2013–2016

**DOI:** 10.1016/j.virs.2022.05.005

**Published:** 2022-05-26

**Authors:** Ting Zhang, Jing Li, Yong-Zhong Jiang, Jun-Qiang Xu, Xu-Hua Guan, Li-Qiang Wang, Jie Chen, Yi Liang

**Affiliations:** a Hubei Key Laboratory of Cell Homeostasis, College of Life Sciences, Wuhan University, Wuhan, 430072, China; b Hubei Provincial Center for Disease Control and Prevention, Wuhan, 430079, China

**Keywords:** Group A human rotavirus (RVA), Prevalence, Phylogenetic analysis, Evolution, Hubei Province

## Abstract

Group A human rotaviruses (RVAs) annually cause the deaths of 215,000 infants and young children. To understand the epidemiological characteristics and genetic evolution of RVAs, we performed sentinel surveillance on RVA prevalence in a rotavirus-surveillance network in Hubei, China. From 2013 to 2016, a total of 2007 fecal samples from hospital outpatients with acute gastroenteritis were collected from four cities of Hubei Province. Of the 2007 samples, 153 (7.62%) were identified positive for RVA by real-time RT-PCR. RVA infection in Hubei mainly occurred in autumn and winter. The highest detection rate of RVA infection was in 1–2 years old of outpatients (16.97%). No significant difference of RVA positive rate was observed between females and males. We performed a phylogenetic analysis of the G/P genotypes based on the partial *VP7/VP4* gene sequences of RVAs. G9P[8] was the most predominant strain in all four years but the prevalence of G2P[4] genotype increased rapidly since 2014. We reconstructed the evolutionary time scale of RVAs in Hubei, and found that the evolutionary rates of the G9, G2, P[8], and P[4] genotypes of RVA were 1.069 ​× ​10^−3^, 1.029 ​× ​10^−3^, 1.283 ​× ​10^−3^ and 1.172 ​× ​10^−3^ nucleotide substitutions/site/year, respectively. Importantly, using a molecular clock model, we showed that most G9, G2, P[8], and P[4] genotype strains dated from the recent ancestor in 2005, 2005, 1993, and 2013, respectively. The finding of the distribution of RVAs in infants and young children in Hubei Province will contribute to the understanding of the epidemiological characteristics and genetic evolution of RVAs in China.

## Highlights

• A four-year study of sentinel surveillance program of RVAs was performed in Hubei, China.
• The key population of rotavirus infection is 1–2 years old of outpatients with acute gastroenteritis.
• G9P[8] was the most predominant strain between 2013 and 2016.
• The estimating time to the most recent common ancestor for the G9 genotype based on partial *VP7* gene was 46 years.
• RVA distribution in Hubei Province contributes to the understanding of the epidemiological characteristics of RVAs in China.

## 1 Introduction

Rotaviruses are double-stranded RNA viruses in the family *Reoviridae*. The viruses are packaged into a triple-layer capsid and divided into seven major groups based on *VP6* gene, from groups A to G. Two proteins in the outermost layer of the viruses, VP4 and VP7, are essential for virulence, host specificity, and neutralizing antibody responses (Matthijnssens et al., 2011), and defined the P and G genotypes, respectively (Iturriza-Gómara et al., 2004). A total of thirty-six G genotypes and fifty-one P genotypes have been identified in humans and other animals so far (https://rega.kuleuven.be/cev/viralmetagenomics/virus-classification/rcwg). Five genotype combinations, G1P[8], G2P[4], G3P[8], G4P[8] and G9P[8], are prevalent in humans (Iturriza-Gómara et al., 2011; Matthijnssens et al., 2011; Santos and Hoshino, 2005). Uncommon combinations, such as G8P[4], G10P[8], and G12P[6], have also been reported in recent years (Durmaz et al., 2014; Gauchan et al., 2013; Oluwatoyin et al., 2012; Pietsch et al., 2009; Weinberg et al., 2012).

Among rotaviruses, group A human rotaviruses (RVAs) are the main cause of acute gastroenteritis among children and annually cause 215,000 deaths of infants and young children worldwide (Tate et al., 2016). Even in developed countries, RVAs are also a major cause of morbidity and levy a great financial burden (Charles et al., 2006). From 1994 to 2014, 40% of diarrhea-related hospitalizations and 30% of diarrhea-related outpatients among children under 5 years old in China were caused by RVAs (Wu et al., 2016). The great diversity of predominant genotypes of RVAs may affect the efficacy of rotavirus vaccination programs (Akran et al., 2010; Aminu et al., 2010; Arista et al., 2006; Glass et al., 2006; Todd et al., 2010). Because of the significant fluctuation of circulating genotypes of RVAs in different research periods and populations, continued surveillance programs in the pre- and post-vaccination eras have been carried out. Such programs can provide important information by monitoring the disease burden of RVA, identifying the temporal changes in circulating genotypes and assessing the effectiveness of existing vaccines (da Silva Soares et al., 2014; Hull et al., 2011; Jin et al., 2008; László et al., 2012; Pukuta et al., 2014; Stupka et al., 2012). Monitoring RVA genotypes detected from diarrhea-related patients will help identify viral strains more effectively, especially imported strains and zoonotic strains, in populations with large-scale vaccination programs.

Here, we performed a four-year study of sentinel surveillance program of RVAs in Hubei, China. We assessed the prevalence of G and P genotypes from RVA strains identified in this program and collected baseline data about circulating genotypes before the implementation of RVA vaccination program. We found that the key population of rotavirus infection was 1–2 years old of outpatients. Our findings could be exploited to understand the epidemiological characteristics and genetic evolution of RVAs in China.

## 2 Materials and methods

### 2.1 Study populations

This active surveillance was conducted in the gastrointestinal clinics of sentinel hospitals in four cities across Hubei Province (Xiangyang, Yichang, Enshi, and Hanchuan). All outpatients visiting the clinics with symptoms of diarrhea (≥ 3 loose or watery stools within 24 ​h), vomiting or fever were included. The epidemiologic settings of this study for RVA infection included people of all ages that meet the case definition. From April 2013 to December 2016, a total of 2,007 stool specimens from outpatients with diarrhea were collected. All the specimens were kept frozen until testing.

### 2.2 RNA extraction and real-time RT-PCR

Stool specimens were diluted 1:10 with 1 ​× ​PBS (pH 7.4). The suspension was centrifuged at 3000×*g* for 30 ​min after shaking. Viral RNA was then extracted from the supernatant using a magnetic bead prefilled DNA/RNA extraction kit (Tianlong, Xi'an, China) according to the manufacturer's instructions. The extracted viral RNA was immediately stored at −80 ​°C for further use. The viral RNA was detected for rotavirus using the AgPath-ID one-step RT-PCR kit (Thermo Fisher Scientific, Foster City, CA) on an ABI 7500 real-time PCR platform (Applied Bioststems, Foster City, CA). The previously published primers and probes were used (Mijatovic-Rustempasic et al., 2013). The reaction mixture (25 ​μL) consisted of 0.4 ​μmol/L of each of two primers and 0.2 ​μmol/L of TaqMan Probe.

### 2.3 Nucleotide DNA sequencing and genotyping analysis

The viral dsRNA was subjected to reverse transcription using a PrimeScript II First-Strand cDNA Synthesis Kit (Takara, Dalian, China). The complete or partial *VP7* and *VP4*-coding genes of RVA were amplified using the primers, as previously described (Iturriza-Gómara et al., 2004; Simmonds et al., 2008). The PCR products were sequenced by Sangon Biotechnology (Shanghai, China). The nucleotide sequences for *VP4* and *VP7* obtained in this article were submitted to the GenBank database with accession codes MG788354 to MG788515 and MG788516 to MG788633, respectively (Supplementary Table S1).

Homology analysis of the RVA strains was performed using Meg-Align in Lasergene 7.0 (http://www.dnastar.com) (DNASTAR, Madison, WI). The G and P genotypes of RVA were determined from partial *VP7* and *VP4* sequences (452 and 572 bp) by phylogenetic analysis with MEGA 6.0 (Tamura et al., 2013) using the maximum likelihood method with 1000 bootstrap replicates. The models T92 ​+ ​G ​+ ​I and GTR ​+ ​G ​+ ​I were applied for the G and P genotypes of RVA, respectively. The phylogenetic trees were viewed with FigTree 1.4.3 (http://tree.bio.ed.ac.uk/software/figtree/). G and P genotype reference strains with established genotypes were selected from GeneBank (Kiseleva et al., 2018; Zhao et al., 2021).

### 2.4 Evolutionary analyses of RVA

Using the Akaike information criterion, the best-fit models of nucleotide substitutions were selected by the jModelTest program Version 0.1.1 (Posada, 2008). The time to the most recent common ancestor and the rate of nucleotide substitution for RVA were estimated by the Bayesian Markov chain Monte Carlo (MCMC) method using BEAST software Version 1.8.1 (Drummond et al., 2012). The best-fit model of nucleotide substitution was GTR ​+ ​G for both the *VP7* and *VP4* genes. Under an uncorrelated exponential derivation (UCED) model, a Bayesian skyline model for population growth was selected using the BEAST software. MCMC analyses were performed for 100 million generations, sampling each tree every 10,000 steps; the first 10% were discarded as chain burn-in. MCMC convergence and the effective sample size estimates (>200) were checked with the Tracer program Version 1.5. After a 10% burn-in, the maximum clade credibility (MCC) tree was generated by Tree Annotator Version 1.8.1 (http://beast.bio.ed.ac.uk/TreeAnnotator). The phylogenetic tree was viewed with FigTree 1.4.3. *VP7* and *VP4* sequences of G2P[4] RVA strains and G9P[8] RVA strains with clear collection date and genotype information were downloaded. Effective selection is made from the earliest time node (April 2013) to the latest time node (December 2016). The selected reference strains were showed in Supplementary Table S2.

### 2.5 Statistical analysis

Clinical and epidemiological information, including gender, age, seasonality and date of sampling, was collected for all patients. The statistical analyses of different age groups were performed using the chi-square (*χ*^2^) test. A value of *P* ​< ​0.05 indicates statistically significant differences. The data were analyzed by using SPSS Statistics for Windows, Version 19.0 (IBM, Armonk, New York).

## 3 Results

### 3.1 Seasonal characteristics of RVA in Hubei Province

The diarrhea monitoring project started from 2013 was performed in sentinel hospitals in four cities of Hubei Province (Xiangyang, Yichang, Enshi, and Hanchuan) (Fig. 1A). In the first two years, 161 and 313 samples were collected, respectively. From 2015, we added a new sentinel monitoring hospital, and 660 and 873 samples were collected in 2015 and 2016, respectively (Fig. 1B). Of the 2007 acute gastroenteritis outpatients, 153 (7.62%) were RVA-positive by real-time RT-PCR. The detection proportion was 9.32% (15/161), 9.27% (29/313), 6.36% (42/660) and 7.67% (67/873) in 2013, 2014, 2015 and 2016, respectively (Fig. 1B). No significant group difference was observed in the detection proportion for RVAs between the four years (*χ*^2^ ​= ​3.344, *P* ​= ​0.342). Seasonal variation of RVAs prevalence was observed (Fig. 1B). RVA infection in Hubei Province mainly occurred in autumn and winter, the coldest and the driest seasons of the year.

**Fig. 1:**
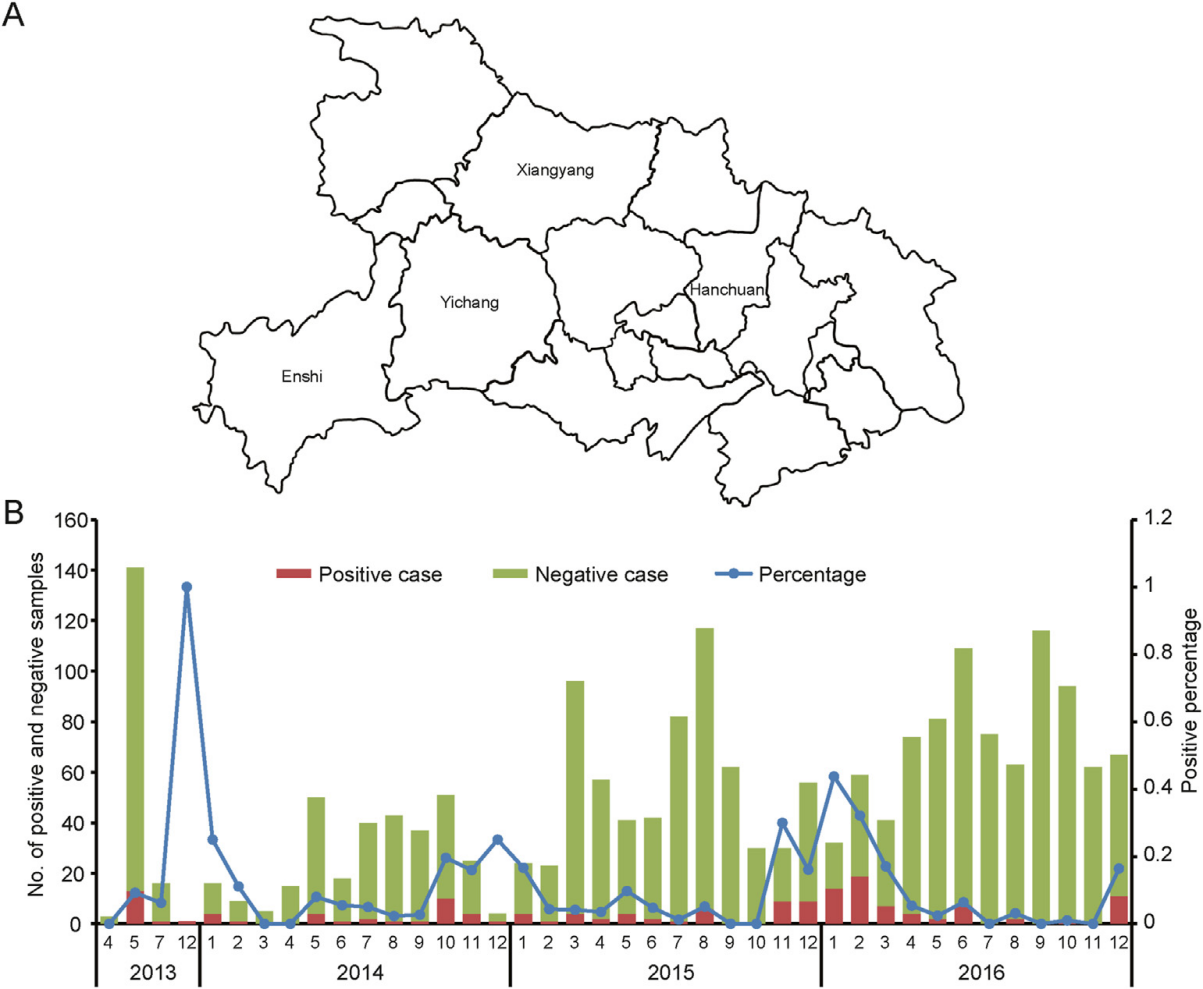
Monthly distribution of RVA detection rate in 2013–2016 in four cities of Hubei Province. **A** The location of the four cities of sentinel hospitals in Hubei Province (Xiangyang, Yichang, Enshi, and Hanchuan). **B** The monthly positive case numbers (red), the monthly negative case numbers (green), and the monthly positive detection percentage (blue).

### 3.2 RVA infection in different age and gender

The RVA-positive patients were comprised of 78 (10.58%) (78/737) children under one year of age, 28 (16.97%) (28/165) children in the 1–2 year age group, 5 (3.94%) (5/127) children in the 2–5 year age group, 3 (2.52%) (3/119) children in the 5–10 year age group, 7 (4.86%) (7/144) youth in the 10–25 year age group, 11 (4.06%) (11/271) persons in the 25–50 year age group, and 21 (4.73%) (21/444) people above 50 years of age (Table 1). The highest detection rate of RVA infection was found in 1–2 years old of outpatients (16.97%) (Table 1). By contrast, the age group with the lowest detection proportion for RVAs was the 5–10 year age group. A significant difference was observed in the detection proportion for RVAs among these age groups (*χ*^2^ ​= ​30.123, *P* ​< ​0.001) (Table 1). Of the 153 RVA-positive samples, 58 samples (37.91%) were from females, and the remaining 95 samples (62.09%) were from males. No significant group difference was observed in the detection proportion for RVAs between females and males (Table 1).

**Table 1:** RVA infection in different age and gender.

Feature	Number of detections	Number of positives (%)	*χ*^2^	*P*
Gender			1.854	0.173
Male	1141	95 (8.33)		
Female	866	58 (6.70)		
Age (year)			30.123	<0.001
0–1	737	78 (10.58)		
1–2	165	28 (16.97)		
2–5	127	5 (3.94)		
5–10	119	3 (2.52)		
10–25	144	7 (4.86)		
25–50	271	11 (4.06)		
50∼	444	21 (4.73)		

### 3.3 Distribution of G and P genotypes

RVA-positive samples identified by real-time RT-PCR were further amplified by conventional RT-PCR to obtain the partial sequences of the *VP7* and *VP4* genes for genotyping. Among the 153 RVA-positive samples, a total of 148 specimens were successfully amplified and assigned to phylogenetic analysis. Based on the phylogenetic analysis, the G genotype RVAs identified in this study were clustered into five genotypes: G9, G2, G1, G3, and G4 (Fig. 2) while the P genotype RVAs were clustered into P[8], P[4], and P[6] genotypes (Fig. 3). Genotype G9 accounted for 67% of RVA G types, followed by G2 (20%), G1 (5%), G3 (4%), G4 (1%), and unidentified G genotypes (3%). The top two P genotypes were P[8] (78%) and P[4] (18%), while the rest were P[4] (18%), P[6] (1%), and unidentified P genotypes (3%). The most common genotype combination of RVAs in Hubei Province was G9P[8] (66%), followed by G2P[4] (18%), G1P[8] (5%), G3P[8] (4%), G2P[8] (2%), G9P[4] (1%), and G4P[6] (1%).

**Fig. 2:**
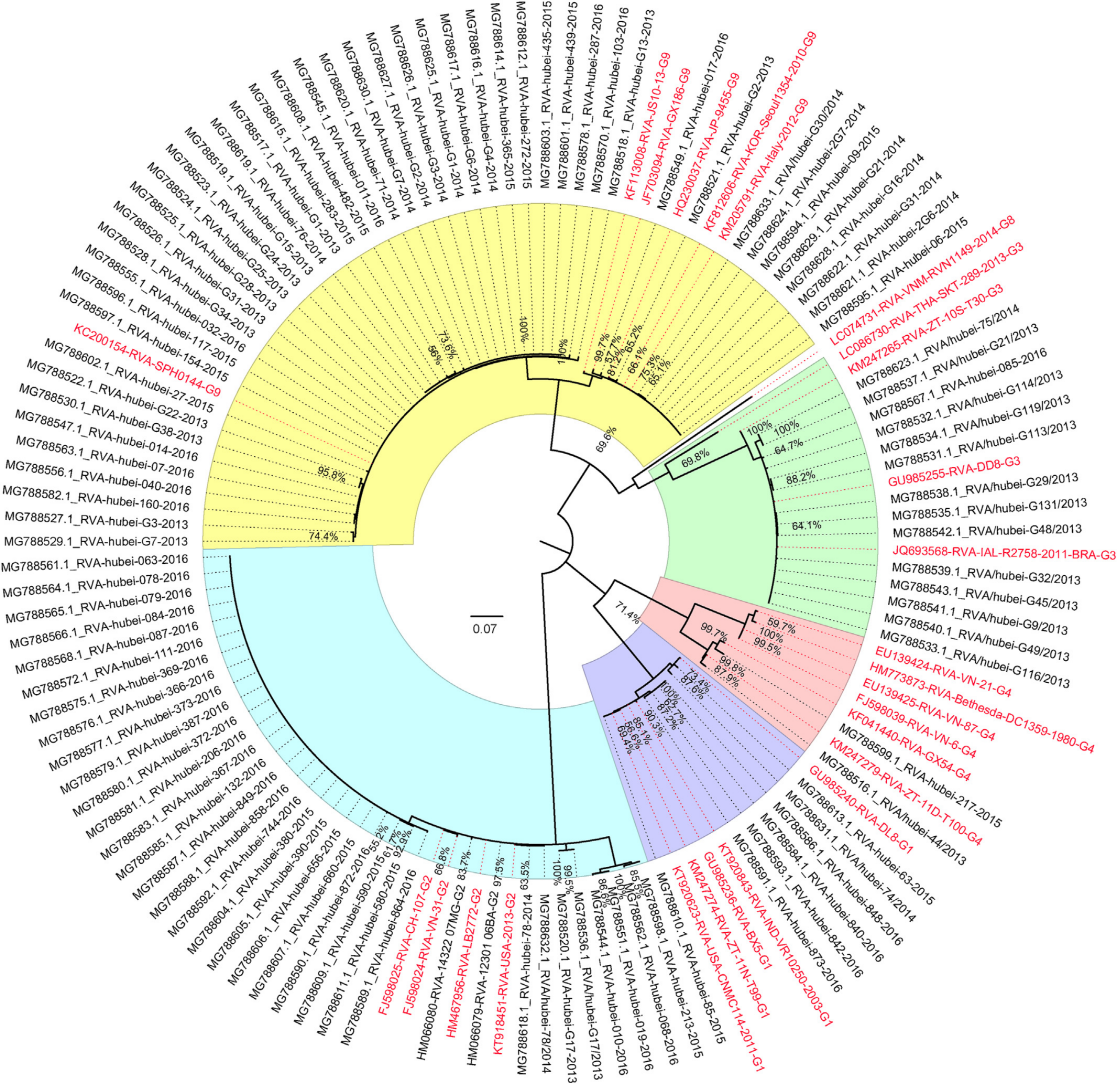
Phylogenetic tree of G genotypes of RVA based on the partial *VP7* gene sequences. Nucleotide sequences were analyzed by using the maximum likelihood method under the T92 ​+ ​G ​+ ​I nucleotide substitution model by bootstrapping with 1000 replicates. The scale bar indicates nucleotide substitutions per site. The taxon names in black are those of the RVA strains detected in Hubei Province, China, in 2013–2016. Sequences of the various G genotypes of reference RVA strains were obtained from GenBank. The reference sequence is shown in red. The yellow, blue, green, purple, and red blocks represent the G9, G2, G3, G1, and G4 genotypes in Hubei Province, respectively.

**Fig. 3:**
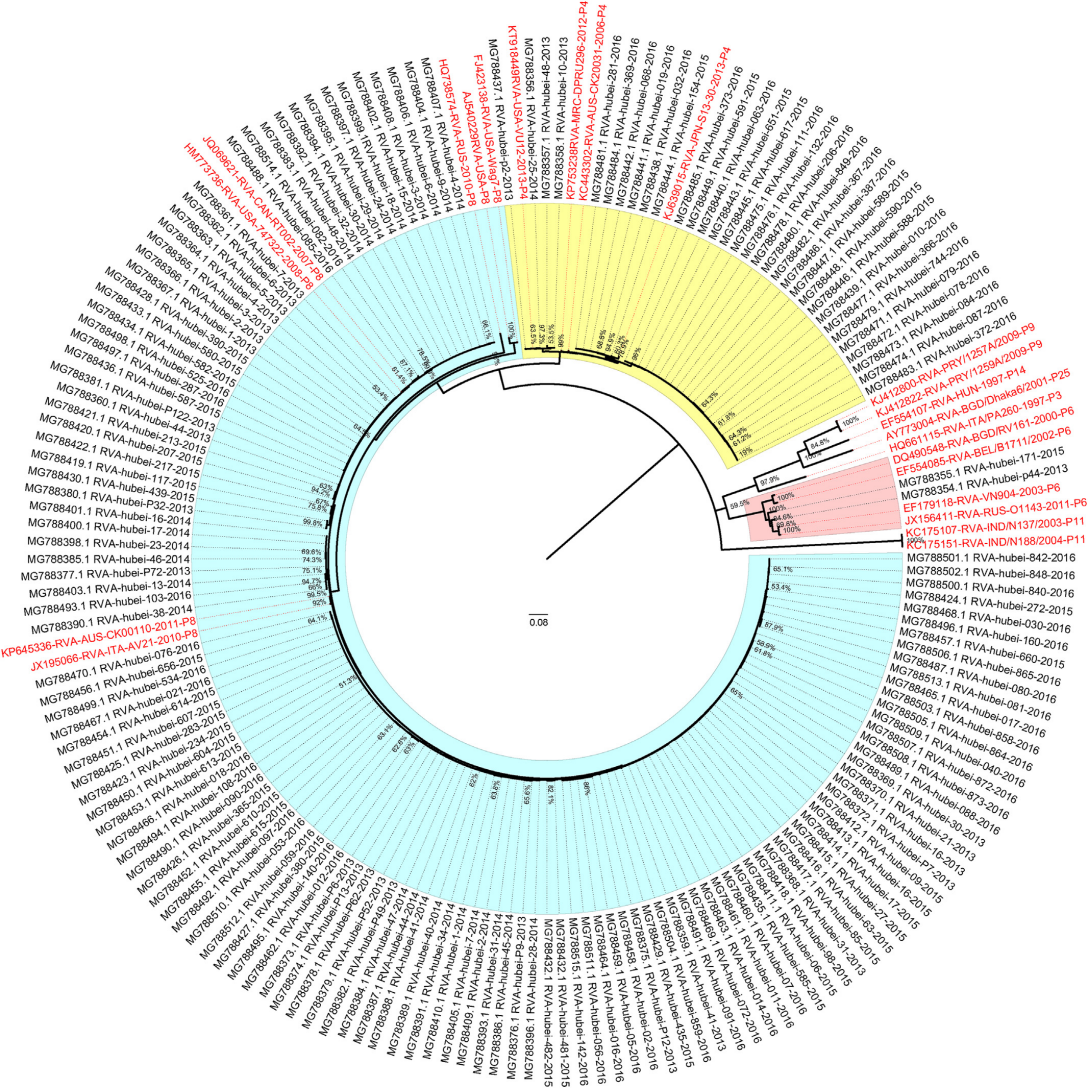
Phylogenetic tree of P genotypes of RVA based on the partial *VP4* gene sequences. Nucleotide sequences were analyzed by using the maximum likelihood method under the GTR ​+ ​G ​+ ​I nucleotide substitution model by bootstrapping with 1000 replicates. The scale bar indicates nucleotide substitutions per site. The taxon names in black are those of the RVA strains detected in Hubei Province, China in 2013–2016. Sequences of the various G genotypes of reference RVA strains were obtained from GenBank. The reference sequence is shown in red. The blue, yellow, and red blocks represent the P[8], P[4], and P[6] genotypes in Hubei Province, respectively.

### 3.4 Distribution of G and P genotypes by age and year

**Fig. 4:**
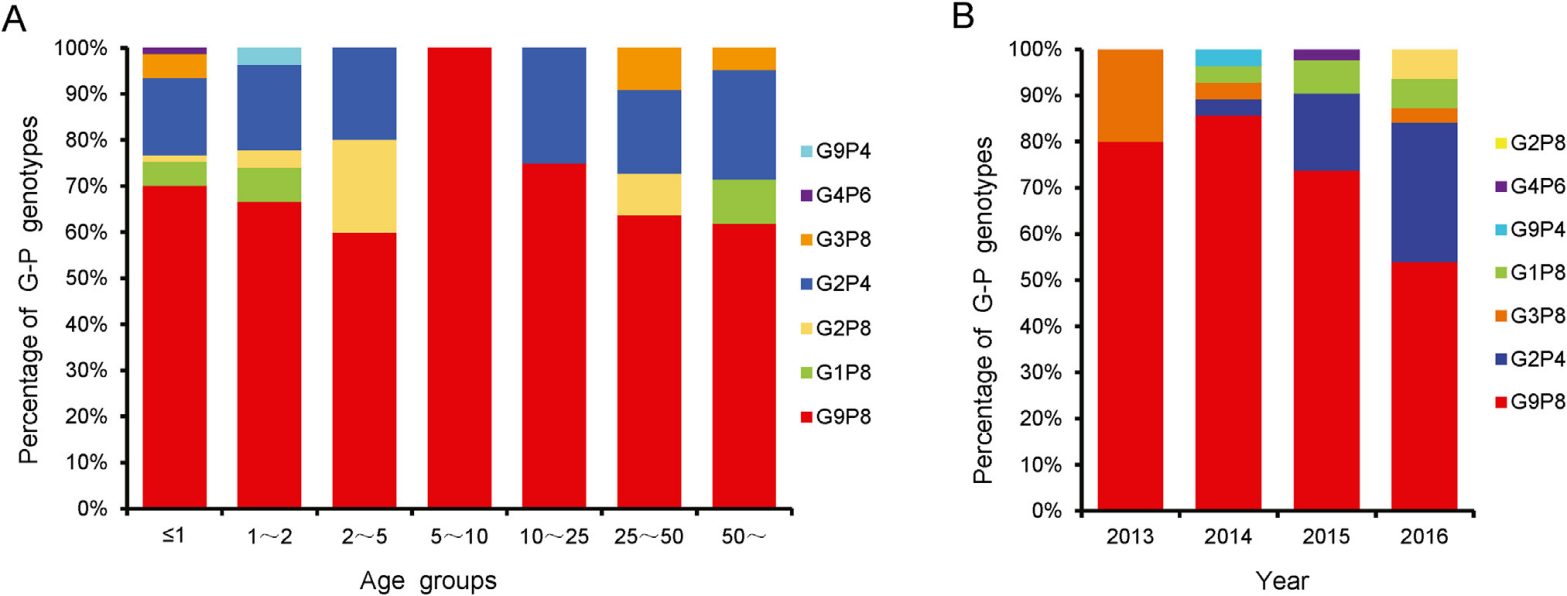
The distribution of G-P combination genotypes in different age groups (**A**) and different years (**B**).

We further studied the distribution of G and P genotypes by age and year (Fig. 4). Seven G-P combination genotypes were found in seven different age groups (Fig. 4A). As shown in Fig. 4A, G9P[8] was the only genotype found in the 5–10 year age group and the most prevalent G-P combination genotype in the other six age groups, and G2P[4] was the second most prevalent genotype in the other six age groups. Genotype G4P[6] was only found in the 0–1 year age group and genotype G9P[4] was only found in the 1–2 year age group (Fig. 4A). As shown in Fig. 4B, G9P[8] was the most predominant G-P genotype in all four years but its proportion decreased year by year. G2P[4] was first identified in 2014 and there was a rapid increase in the prevalence in the following two years (Fig. 4B). G9P[4] and G4P[6] only occurred in 2014 and 2015, respectively. Notably, a new genotype, G2P[8] appeared in 2016 (Fig. 4B). In the four years of testing, it is particularly noted that genotype G2P[8] was detected for the first time in 2016, the most recent year (Fig. 4B). Therefore, we should closely follow and monitor genotypes G2P[8], G2P[4], and G9P[8] in future studies.

### 3.5 Genetic evolution of RVAs in Hubei Province

**Fig. 5:**
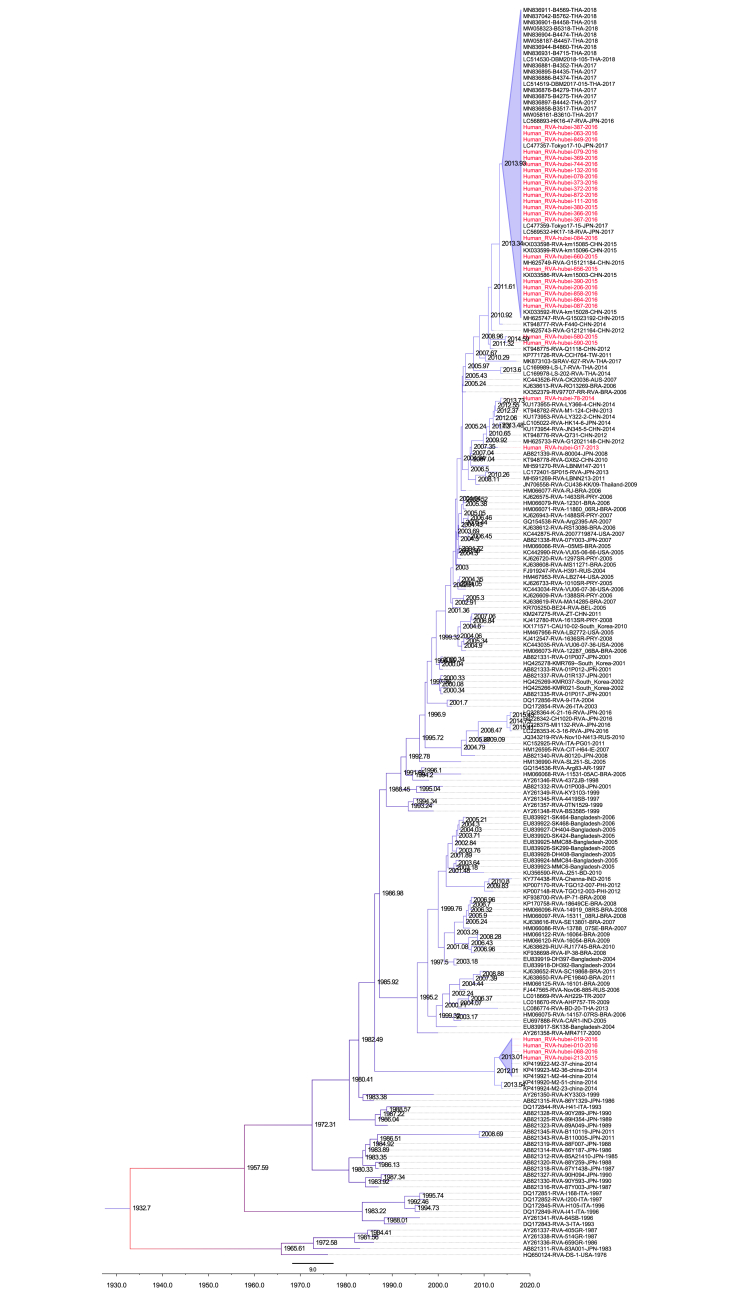
MCC tree based on the nucleotide sequences of *VP7* genes of G2P[4] RVA strains. The Bayesian evolutionary analysis was performed based on 172 selected *VP7* gene sequences and 31 sequences isolated in this study. The trees were estimated with the GTR ​+ ​G nucleotide substitution model, a UCED model, and the Bayesian skyline analysis as a tree prior. The taxon names in red are those of the RVA strains detected in Hubei Province, China in 2013–2016.

**Fig. 6:**
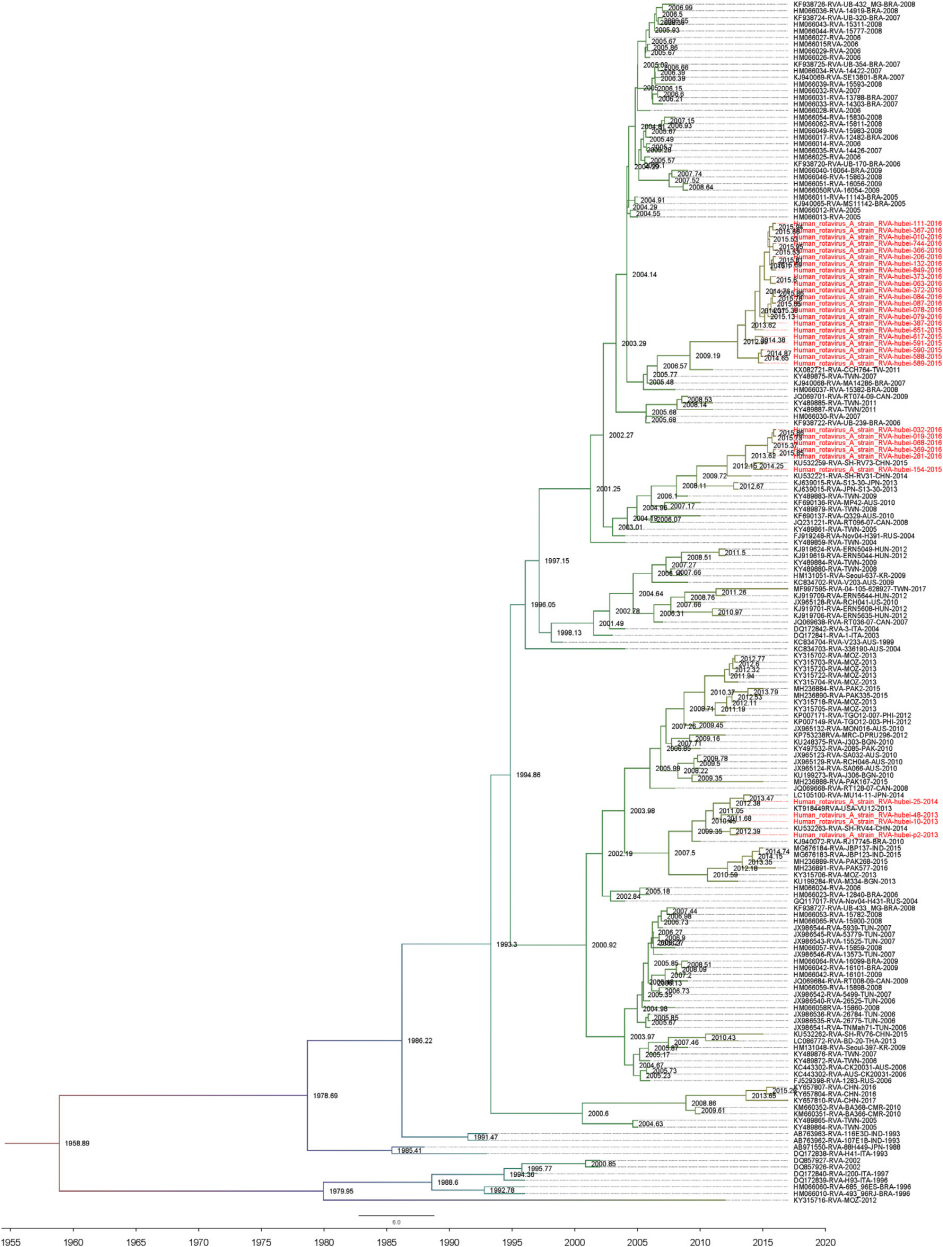
MCC tree based on the nucleotide sequences of *VP4* genes of G2P[4] RVA strains. The Bayesian evolutionary analysis was performed based on 148 selected *VP4* gene sequences and 32 sequences isolated in this study. The trees were estimated with the GTR ​+ ​G nucleotide substitution model, a UCED model, and the Bayesian skyline analysis as a tree prior. The taxon names in red are those of the RVA strains detected in Hubei Province, China in 2013–2016.

Since G2P[4] and G9P[8] were the main popular G-P combination genotypes in year 2015–2016 (Fig. 4), we further perform the Bayesian evolutionary analysis based on *VP7* and *VP4* gene sequences of G2P[4] (Fig. 5, Fig. 6) and G9P[8] genotypes (Supplementary Figs. S5 and S6). The root-to-tip regression analysis (Rambaut et al., 2016) based on the partial *VP7* and *VP4* gene sequences collected in this study (Supplementary Table S1) and reference strains (Supplementary Table S2) were performed to obtain sequences for Bayesian evolutionary analysis (Supplementary Figs. S1–S4).

Bayesian evolutionary analysis was performed based on 172 selected *VP7* gene sequences of G2P[4] and 31 sequences isolated in this study. The earliest evolutionary ancestor of the G2 genotype strains derived from 1933 and the significant time nodes for differentiation were in 1958, 1966, 1972, and 1986 (Fig. 5). As shown in Fig. 5, most G2 genotype strains from Hubei Province dated from the recent ancestor in 2005. Only a few G2 strains from Hubei Province had the most recent ancestor in 2013 (Fig. 5). Bayesian evolutionary analysis based on 148 selected *VP4* gene sequences of G2P[4] and 32 sequences isolated in this study showed that the P[4] strains from Hubei Province were located in three clusters and the earliest ancestor could be dated back to 1959 (Fig. 6). Most P[4] genotype strains from Hubei Province derived from the recent ancestor in 2013 and two other clusters of P[4] strains from Hubei Province had the most recent ancestor in 2014 and 2010, respectively. The results of evolutionary history imply that RVA strains of the same genotype of Hubei Province from 2013 to 2016 could have different evolutionary sources.

The MCC tree of the G9 genotype (Supplementary Fig. S5) using the Bayesian skyline demographic model shows that this genotype of RVA strains in Hubei Province had different evolutionary time nodes, located in two different clusters. Most G9 genotype strains from Hubei Province were derived from the prior ancestor in 2005 and differentiated into five lineages, and some G9 genotype strains of Hubei Province dated from the recent ancestor in 2011 (Supplementary Fig. S5). The P[8] genotype of RVA strains in Hubei Province was located in cluster-specific MCC trees, and the P[8] gene of RVA from Hubei Province derived from 1993 gradually evolved into two lineages (Supplementary Fig. S6).

The molecular clock model, as a UCED model, was used to calculate both the rates of evolution and the time to the most recent common ancestor of the G9, G2, P[8] and P[4] genotypes based on the partial *VP7* and *VP4* gene sequences (Table 2). The average times to the most recent common ancestor for the partial *VP7* gene of the G9 genotype and the partial *VP4* gene of the P[8] genotype were 46 years (range 23, 78) and 107 years (range 52, 183), respectively, which dated the most recent ancestor back to 1975 and 1913 (Table 2). The evolutionary rates (the nucleotide substitution rates) of the G9, G2, P[8] and P[4] genotypes of RVA were 1.069 ​× ​10^−3^, 1.029 ​× ​10^−3^, 1.283 ​× ​10^−3^ and 1.172 ​× ​10^−3^ nucleotide substitutions/site/year, respectively, using the UCED clock, and the nucleotide substitution rate of the G9 genotype of RVA was similar to that of the RVA G2 genotype.

**Table 2:** Nucleotide substitution rates and divergence times for the G and P genotypes of RVA in Hubei Province, China.

Gene	Genotype	Nucleotide substitution rate (substitutions/site/year)	TMRCA	
			No. of years	Year (range)
*VP7*	G9	1.069 ​× ​10^−3^ (7.102 ​× ​10^−4^, 1.445 ​× ​10^−3^)	46 (23, 78)	1975 (1943, 1998)
	G2	1.029 ​× ​10^−3^ (7.252 ​× ​10^−4^, 1.354 ​× ​10^−3^)	84 (49, 134)	1933 (1883, 1968)
*VP4*	P[8]	1.283 ​× ​10^−3^ (9.190 ​× ​10^−4^, 1.651 ​× ​10^−3^)	107 (52, 183)	1913 (1837, 1968)
	P[4]	1.172 ​× ​10^−3^ (8.460 ​× ​10^−4^, 1.486 ​× ​10^−3^)	58 (33, 94)	1959 (1923, 1984)

95% highest posterior density (HPD) lower, 95% HPD upper; the nucleotide substitution rate is the mean rate for the three individual determinations. UCED, uncorrelated exponential deviation clock. TMRCA, time to most recent common ancestor. The values in parentheses are the 95% HPDs.

## 4 Discussion

In this article, we performed the epidemiology of RVAs on the 2,007 fecal samples from outpatients with acute gastroenteritis, in Hubei, from 2013 to 2016. Partial sequencing of the *VP7* and *VP4* genes revealed the diverse G/P genotypes and alternative prevalence genotypes of RVAs circulating in Hubei. We also analyzed the possible relevance of RVA infections and patient ages as well as seasons. Additionally, we performed the analysis of the evolutionary time scale for G2P[4] and G9P[8] of RVAs. This study provides basal data for understanding the molecular epidemiology and evolution of RVAs.

The epidemiology of RVA-associated diseases depends on not only the social and economic conditions of the study group but also on climatic differences (Cunliffe et al., 1998; Hacimustafaoğlu et al., 2011; Iturriza-Gómara et al., 2011). In China, RVA infections primarily occur between October and the next March (Duan et al., 2009; Fang et al., 2005; Xiao et al., 2014). RVA infections usually have a lower incidence rate in spring and summer, with an increased rate in late autumn (Dian et al., 2017). In this research, the epidemic seasons occurred during the cold months between October and the next February, consistent with the common characteristics of RVAs. The seasonal peak of onset is in winter, especially in November (Wang et al., 2007, 2009; Yu et al., 2019). We found that the highest positive proportion of RVA was in 1–2 years old children. Most literature suggests that the highest detection rate of RVA is in children under 2 years old (Duan et al., 2009; Jin et al., 2009); our study is consistent with this conclusion. No association was found between gender and the detection rate of RVA.

In China, serotype G1 was the main strain before 2000, but G3 has been the predominant strain since 2000 (Li et al., 2014). From 1994 to 2012, the genotypes G1, G3, and G2 were the most common G genotypes, followed by G9 but not G4 (Duan et al., 2009). In 1998, G9 was first discovered in Yunnan Province of China, and its prevalence rate increased during subsequent years (Li et al., 2014). By 2011, G9 had replaced the G1 and G3 strains, and has become the most common genotype in Wuhan (Wang et al., 2014). During 1994 to 2012, little variation in P genotypes was found in China and serotype P[8] is the most common strain, followed by P[4] (Li et al., 2014). Comparatively, P[9] and P[10] strains were reported frequently in the southern and northern regions of China, respectively (Li et al., 2014). Before 2011, G3P[8] was predominant, followed by G1P[8], while G1 became the predominant genotype in 2011, followed by G1P[8] in 2012 (Wang et al., 2011). From 2009 to 2015, G9P[8] became the predominant strain in China, and the main five combinations were G9P[8], G3P[8], G1P[8], G2P[4], and G3P[4] (Yu et al., 2019). Two studies also reported that G9P[8] was the most common genotype combination circulating in the local population of Beijing, Gansu, and Kunming (Zhang et al., 2015, 2017). Our data showed that G9 and P[8] were the dominant genotypes in Hubei Province from 2013 to 2016, which was consist with data in other regions of China. Because of the segmented feature of the RVA genome, the genes of *VP7* and *VP4* can segregate in an independent manner which contributed to the diversity and recombination of RVA genotypes, resulting in a large number of different combinations of strains. Our molecular surveillance of RVA infections is of significance for comparing the changes in RVA strains and provides an important reference for the prevention and vaccine development.

In this research, the time-scale evolutionary analysis based on partial *VP7*/*VP4* genes of were performed using the UCED clock. The evolutionary rates of the G9, G2, P[8] and P[4] genotypes of RVA were 1.069 ​× ​10^−3^, 1.029 ​× ​10^−3^, 1.283 ​× ​10^−3^ and 1.172 ​× ​10^−3^ nucleotide substitutions/site/year, respectively, similar to those of most RNA viruses, which evolve at a rate of approximately 1 ​× ​10^−3^ nucleotide substitutions/site/year (Duffy et al., 2008). We then compared the substitution rates of *VP7* and *VP4* sequences from this study with reported data. The evolutionary rate of the *VP7* gene of RVA G2P[4] and G9P[8] strains was 1.029 ​× ​10^−3^ and 1.069 ​× ​10^−3^ nucleotide substitutions/site/year, similar to the previous observation (1.18 ​× ​10^−3^ nucleotide substitutions/site/year for G2P[4], Agbemabiese et al., 2016; 1.87 ​× ​10^−3^ nucleotide substitutions/site/year for G9P[8], Matthijnssens et al., 2010). The evolutionary rate of the *VP4* gene of RVA G2P[4] strains was 1.172 ​× ​10^−3^ nucleotide substitutions/site/year, while the evolutionary rate of *VP4* gene calculated from the *VP4* sequences of G2P[4] strains and non G/P genes of DS-1-like RVA strains was 8 ​× ​10^−4^ nucleotide substitutions/site/year (Agbemabiese et al., 2016). In our study, the evolutionary rate of *VP4* gene of RVA G2P[4] strain is remarkably faster than the previous observation, therefore we should pay attention to the evolutionary rate of P[4] genotype in order to detect the evolutionary trend of rotaviruses.

Herein, the phylogenetic analysis and evolutionary history reconstruction showed that RVA strains from Hubei Province differentiated at different time points. Although the RVA from Hubei Province had a common ancestor in an earlier time, we observed that the genes of G9, G2, P[8] and P[4] divided into different evolution directions gradually. More importantly, the same genotype epidemic strains of RVA in the same year could have different evolutionary paths and sources. Therefore, we should not only monitor the genotypes of RVA but also pay close attention to the intergenotype genetic differences by evolution. Our finding of the distribution of RVAs in infants and young children in Hubei Province will contribute to the understanding of the epidemiological characteristics and genetic evolution of RVAs in China.

The World Health Organization (WHO) recommended two licensed rotavirus vaccines, RotaTeq® (RV5, G1–G4, and P[8]) and Rotarix® (RV1, G1P[8]) in 2003, showing high efficacy against homotypic and heterotypic RVA strains in developed countries (Nelson et al., 2008; Leshem et al., 2014). Introduction of rotavirus vaccines could confer partial protection against RVA gastroenteritis (Chandran et al., 2010; Fu et al., 2012; Li et al., 2019), but also impose additional selective pressure on circulating RVA strains, possibly influencing their evolutionary rates. Before the implementation of universal rotavirus vaccination in a country, it is important to understand the status of RVA infections in clinical settings (Nelson et al., 2008). A comparison of the related content research before and after vaccination has not been carried out in Hubei Province so far. Thus, research on this field should be conducted in the future.

## 5 Conclusions

In this study, we performed a four-year study of sentinel surveillance program of RVAs in Hubei Province and found that the key population of rotavirus infection is 1–2 years old of outpatients with acute gastroenteritis. Our data showed that G9P[8] was the most predominant strain in all four years and all G9 genotypes of RVA strains have different evolution trend. This rotavirus surveillance can be used as an entry point to further determine the molecular epidemiological characteristics, major circulating genotypes, evolutionary histories, and evolutionary divergence times of RVAs in Hubei Province.

## Data availability

All the data generated or analyzed during the current study are included in this published article or available from the corresponding author upon request.

## Ethics statement

All institutional and National guidelines about the use of clinical samples were followed. The informed consent has been obtained from all participants and the studies have been approved by the Ethics Committee of the College of Life Sciences, Wuhan University.

## Author contributions

Ting Zhang: Conceptualization, Investigation, Methodology, Validation, Writing ​− ​Original draft preparation, Visualization. Jing Li: Investigation, Methodology, Validation, Formal analysis. Yong-Zhong Jiang: Investigation, Methodology. Jun-Qiang Xu: Investigation, Methodology. Xu-Hua Guan: Investigation, Methodology. Li-Qiang Wang: Formal analysis, Data curation, Validation. Jie Chen: Project administration, Resources. Yi Liang: Supervision, Project administration, Funding acquisition, Conceptualization, Investigation, Methodology, Validation, Writing- Original draft preparation, Writing ​− ​Reviewing and Editing.

## Conflict of interest

The authors declare that they have no conflict of interest.

## Figures and Tables

**Figs1:**
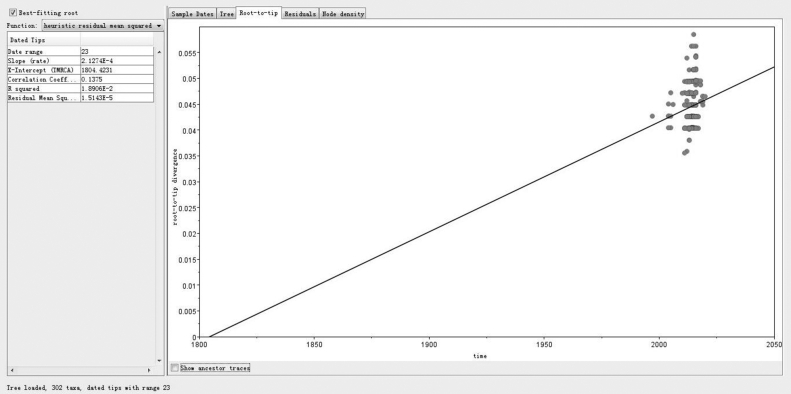


**Figs2:**
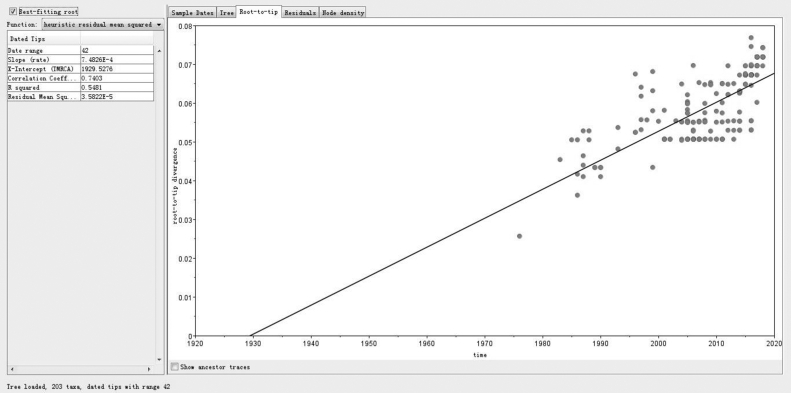


**Figs3:**
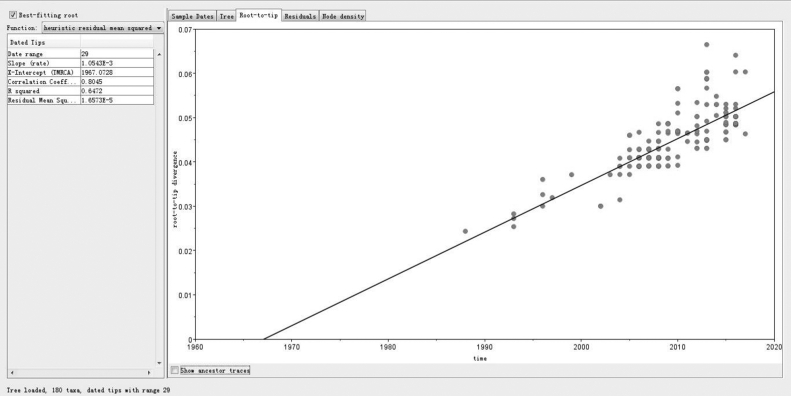


**Figs4:**