# Supplementary material for: Genotype distribution and evolutionary analysis of rotavirus associated with acute diarrhea outpatients in Hubei, China, 2013–2016
Source: Virol Sin. 2022 May 26;37(4):503–12. doi: 10.1016/j.virs.2022.05.005 (PMC9437618; doi:10.1016/j.virs.2022.05.005)
Supplement: Multimedia component 3 [file mmc3.pdf]

Virologica Sinica

Supplementary Data

Genotype Distribution and Evolutionary Analysis of Rotavirus Associated with Acute Diarrhea Outpatients in Hubei, China, 2013–2016

Ting Zhang [a, 1], Jing Li [b, 1], Yong-Zhong Jiang [b], Jun-Qiang Xu [b], Xu-Hua Guan [b], Li-Qiang Wang [a], Jie Chen [a], Yi Liang [a, *]

[a] *Hubei Key Laboratory of Cell Homeostasis, College of Life Sciences, Wuhan University, Wuhan 430072, China*
[b] *Hubei Provincial Center for Disease Control and Prevention, Wuhan 430079, China*

*Corresponding author
Email address: liangyi@whu.edu.cn (Y. Liang)
ORCID: 0000-0002-7349-8300

[1] Ting Zhang and Jing Li have contributed equally.

Supplementary Table S1. List of RVA sequences identified in this study.

Supplementary Table S2. List of the reference sequences from GenBank used in our Bayesian evolutionary analysis.

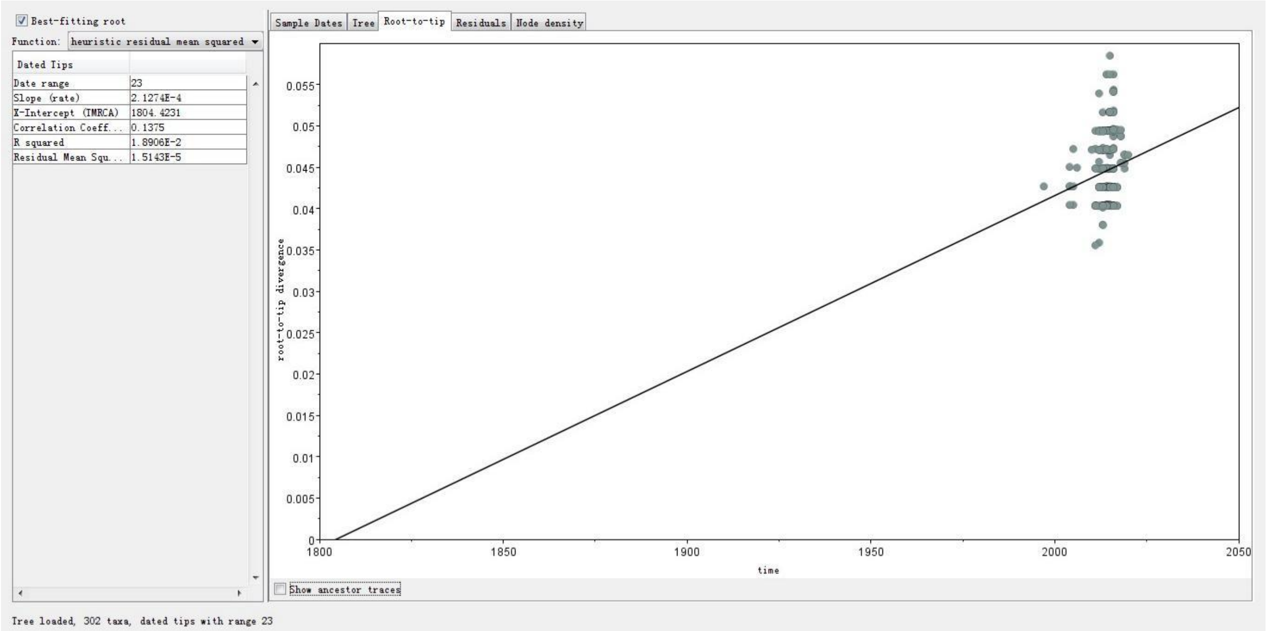

Supplementary Figure S1. The root-to-tip divergence plot of 197 selected *VP7* gene sequences and 105 sequences of G9 RVAs isolated in this study.

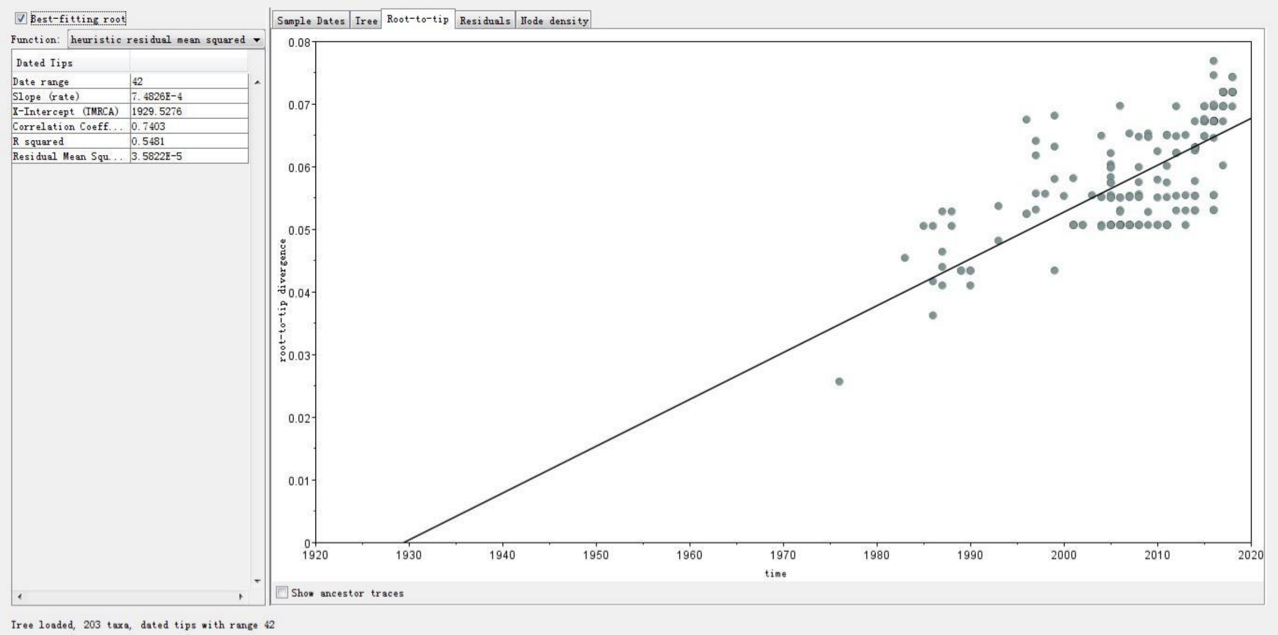

Supplementary Figure S2. The root-to-tip divergence plot of 172 selected *VP7* gene sequences and 31 sequences of G2 RVAs isolated in this study.

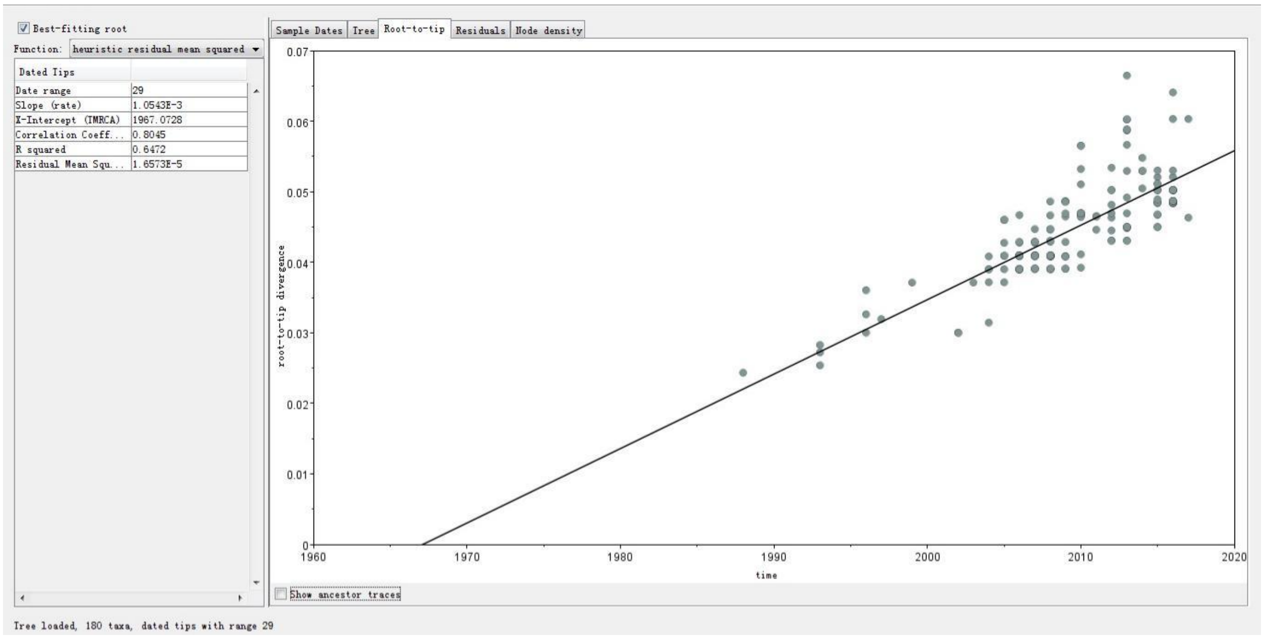

Supplementary Figure S3. The root-to-tip divergence plot of 148 selected *VP4* gene sequences and 32 sequences of P4 RVAs isolated in this study.

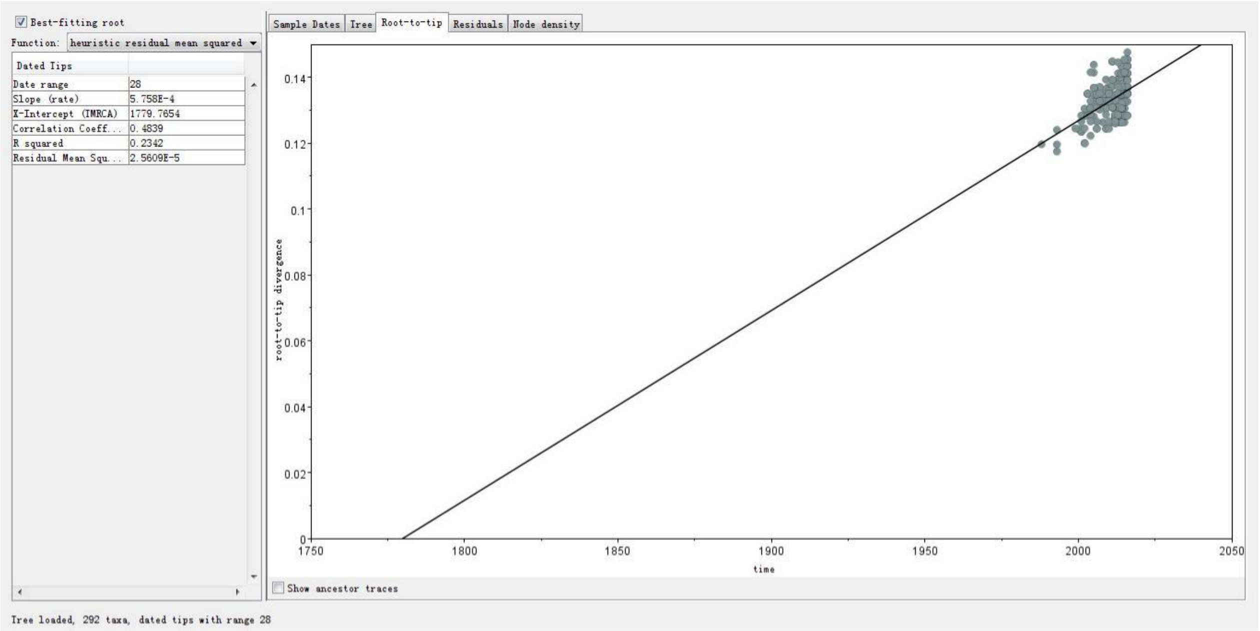

Supplementary Figure S4. The root-to-tip divergence plot of 164 selected *VP4* gene sequences and 128 sequences of P8 RVAs isolated in this study.

Supplementary Figure S5. MCC tree of G9P[8] RVA strains based on *VP7* genes. The Bayesian evolutionary analysis was performed based on 197 selected *VP7* gene sequences and 105 sequences isolated in this study. The trees were estimated with the GTR+G nucleotide substitution model, a UCED model, and the Bayesian skyline plot as a tree prior. The taxon names in red are those of the RVA strains detected in Hubei Province, China in 2013–2016.

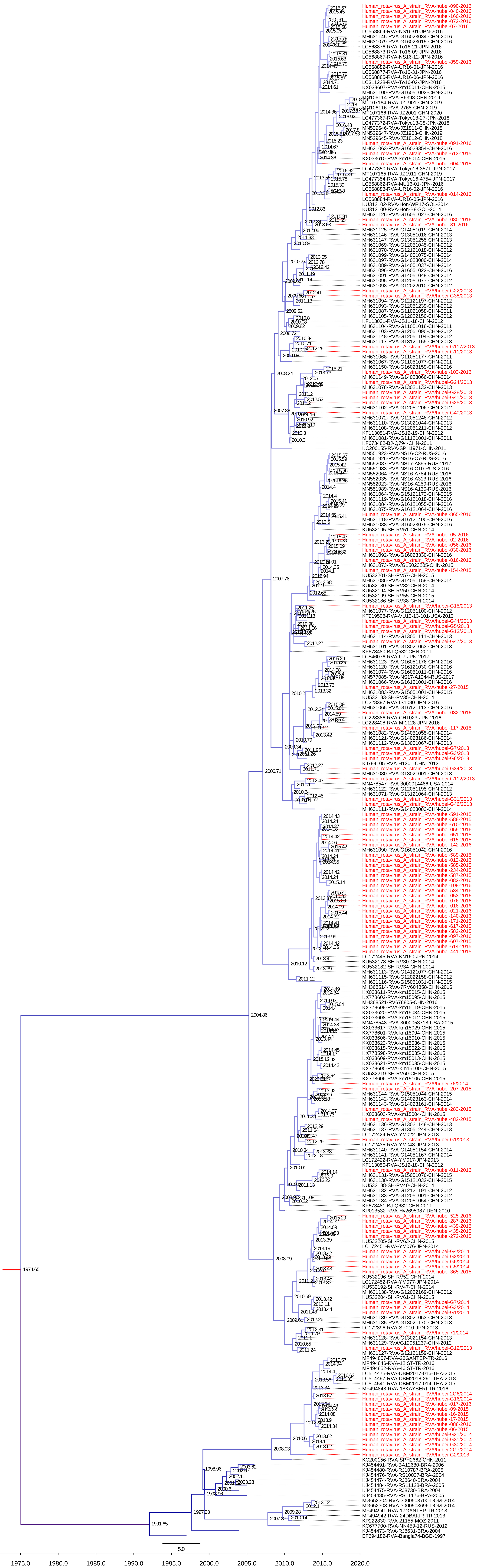

5.0
1975.0
1980.0
1985.0
1990.0
1995.0
2000.0
2005.0
2010.0
2015.0
2020.0

Supplementary Figure S6. MCC tree based on the nucleotide sequences of VP4 genes of G9P[8] RVA strains. The Bayesian evolutionary analysis was performed based on 164 selected *VP4* gene sequences and 128 sequence isolated in this study. The trees were estimated with the GTR+G nucleotide substitution model, a UCED model, and the Bayesian skyline analysis as a tree prior. The taxon names in red are those of the RVA strains detected in Hubei Province, China in 2013–2016.

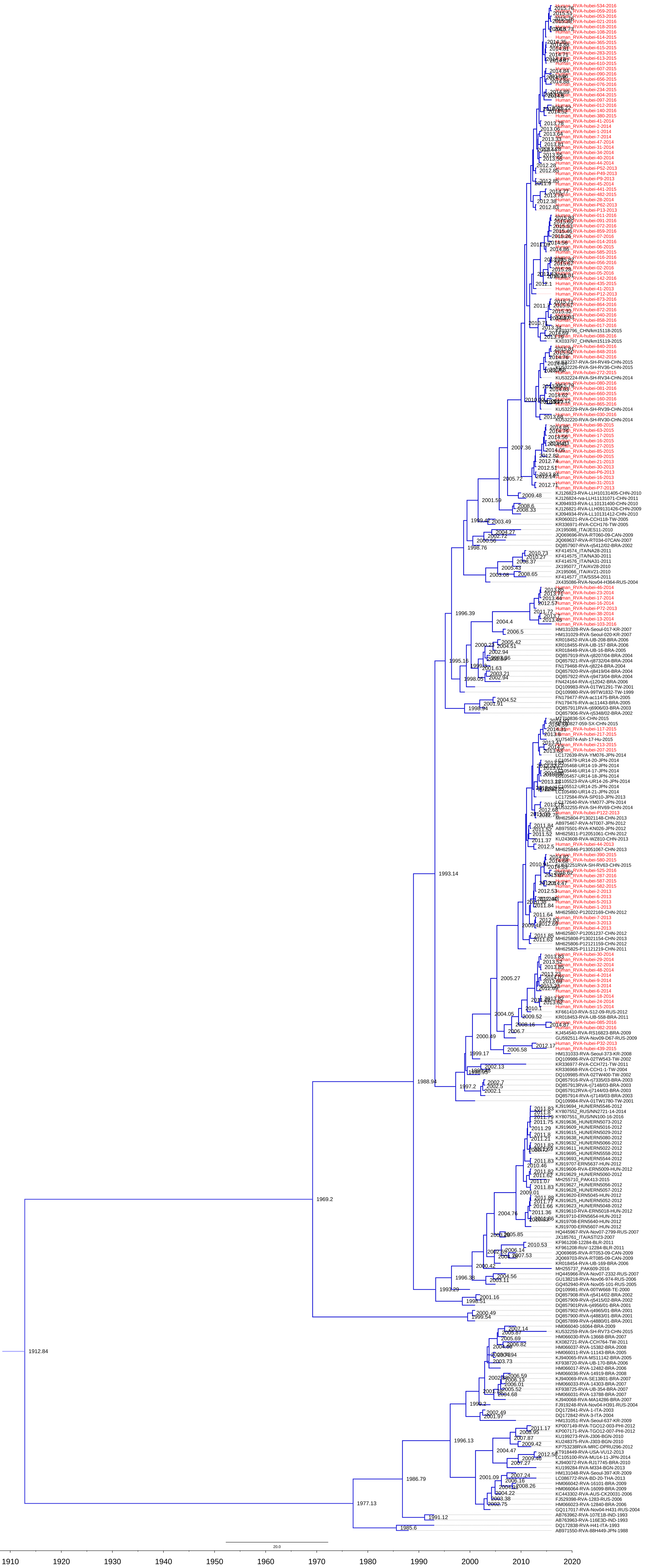
1912.84
1969.2
1977.13
1986.79
1988.94
1993.14
1993.29
1996.13
1996.39
20.0
1910
1920
1930
1940
1950
1960
1970
1980
1990
2000
2010
2020
